# Effect of Probiotics on Respiratory Tract Allergic Disease and Gut Microbiota

**DOI:** 10.3389/fnut.2022.821900

**Published:** 2022-02-22

**Authors:** Jinli Huang, Juan Zhang, Xingzhi Wang, Zenghui Jin, Panpan Zhang, Hui Su, Xin Sun

**Affiliations:** ^1^Department of Pediatrics, Xijing Hospital, The Fourth Military Medical University, Xi'an, China; ^2^Department of Geratology, Xijing Hospital, The Fourth Military Medical University, Xi'an, China

**Keywords:** probiotics, allergic airway inflammation, asthma, allergic rhinitis, gut microbiota

## Abstract

Allergy is a hypersensitivity reaction triggered by specific cell or antibody-mediated immune mechanisms. Allergies have increased in industrialized countries in recent decades. The rise in allergic respiratory diseases such as allergic rhinitis (AR) and allergic asthma (AA) is a potential threat to public health. Searches were conducted using PubMed, Google Scholar and Medline using the following key terms: allergic rhinitis OR asthma AND probiotics, allergic airway inflammation AND immune disorders, probiotics OR gut microbiota AND allergic disease, probiotics AND inflammatory. Studies from all years were included, specifically those published within the last 10 years. Some review articles and their reference lists were searched to identify related articles. The role of microbiota in respiratory allergic diseases has attracted more and more attention. Pieces of evidence suggested that the development of allergic diseases causes a possible imbalance in the composition of the gut microbiota. Compared to colonized mice, germ-free mice exhibit exaggerated allergic airway responses, suggesting that microbial host interactions play an important role in the development of allergic diseases. Probiotics modulate both the innate and adaptive inflammatory immune responses, often used as dietary supplements to provide health benefits in gastrointestinal disorders. Probiotics may serve as immunomodulators and activators of host defense pathways. Besides, oral probiotics can modulate the immune response in the respiratory system. Recently, studies in humans and animals have demonstrated the role of probiotic in RA and AA. To understand the characterization, microbiota, and the potential role of probiotics intervention of AA/AR, this review provides an overview of clinical features of AA and AR, probiotics for the prevention and treatment of AR, AA, changes in gut microbiota, and their mechanisms of action.

## Introduction

Respiratory tract allergic disease includes allergic asthma (AA) and allergic rhinitis (AR). Asthma is one of the most common chronic non-communicable diseases. Approximately 334 million people around the world suffer from asthma (1). Primary characterization of AA is airway inflammation, both reversible airway obstruction and intra-airway hyperresponsiveness. Nasal mucosa swelling, enhanced vascular permeability, mass watery secretions are features of AR. The identity in upper and lower airway makes AA associated with AR. AR is a common allergic airway disease that associated with allergic AA (2). The global prevalence of AR among children was 2~25%, adults were 10~40% (3).

Type I hypersensitivity reactions refer to the acute allergic reaction that takes place after an allergen challenge. The most important humoral player is allergen-specific immunoglobulin E (IgE), which is induced in a T helper-2 (Th2) milieu associated with diseases of the atopy spectrum, such as AR and AA. Allergen-specific IgE is bound *via* its Fc-tail to high-affinity Fc receptors (FcεRI) on mast cells in tissue and basophils in blood. Allergen binding to multiple IgE molecules on mast cells and basophils, so-called IgE crosslinking, will activate these cells and result in degranulation and immediate release of several pre-formed inflammatory mediators, including histamine, leukotrienes, and prostaglandins (4). In addition, mast cells and basophils will release inflammatory cytokines and chemokines (5). In both AA and AR, the inflammatory response involves innate immunity eosinophils, neutrophils, macrophages, mast cells, natural killer cells, γδ-T cells, innate lymphoid cells (ILCs) and dendritic cells (DCs) and adaptive immunity (T and B lymphocytes). Majority of asthma patients are atopic and have an allergic pattern of inflammation in their airways (6). T lymphocytes play a very important role in coordinating the inflammatory response in asthma through the release of specific patterns of cytokines, resulting in the recruitment and survival of eosinophils and in the maintenance of a mast cell population in the airways (7). Mast cells are key effector cells in asthma through their release of multiple bronchoconstrictor mediators, such as histamine, cysteinyl-leukotrienes and prostaglandin (PG)-D2 (8). The role of macrophages in asthma is currently uncertain as they may have pro-inflammatory or anti-inflammatory effects. Macrophages may be activated by allergens *via* low-affinity IgE receptors (9). DC takes up allergens, process them to peptides and migrate to local lymph nodes where they present the allergenic peptides to uncommitted T lymphocytes to programme the production of allergen-specific T cells. Immature DC in the respiratory tract promotes helper Th2 cell differentiation (10). The cytokine thymic stromal lymphopoietin released from epithelial cells in asthmatic patients programmes DC to release chemokines that attract Th2 cells into the airways (11). Eosinophilic inflammation is a characteristic feature of asthmatic airways (12). The mechanisms of neutrophilic inflammation in asthma are uncertain and could be related to the use of high doses of corticosteroids which prolong neutrophil survival in the airways or due to bacterial infection (13). The neutrophilic inflammation in severe asthma may be orchestrated by Th17 cells and increased expression of IL-17A and IL-17F is described in airways of patients with severe asthma (14). Allergic Rhinitis is a Th2-driven, IgE-mediated disease, is characterized by mucosal inflammation, driven by activated immune cells. Mast cells and Th2 cells might decrease epithelial barrier integrity in AR, maintaining a leaky epithelial barrier (15). Recent studies have demonstrated that increased IL-17 levels and Th17 cell number in nasal mucosa and peripheral blood are associated with clinical severity in patients with AR (16). An in-season study involving grass pollen-sensitized patients of allergic rhinoconjunctivitis showed a significant increase in circulating ILC2 numbers (17). ILC2s represent an abundant alternative source of Th2 cytokines and likely serve to amplify and maintain local Th2-driven allergic inflammation (18). The cardinal features of allergic rhinitis include increased allergen-specific IgE concentrations to clinically relevant allergens, IgE-dependent activation of mast cells, and local eosinophilia in target organs. Specific IgE can be synthesized and produced locally by B cells within the respiratory mucosa (19).

AA and AR are IgE mediated responses. Not only is ecological dysbiosis of the airway microbiota associated with AA and AR, but also there may be similar ecological dysbiosis of the gut microbiota (20, 21). The human microbiota starts from the mouth and crosses the esophagus, then the gastric, small intestine, and colon, and finally, reaches the rectum. The distribution surface area of microbiota in the human body is 150–200 m^2^, provides the opportunity for microbial colonization or transient occupation. Bacteria are found in many parts of the human body primarily on the external and internal surfaces, the vast majority of commensal bacteria reside in the colon, about 10^14^ bacteria, followed by the skin, about 10^12^ bacteria. Less than 10^12^ bacteria populate the rest of the body (22).

Asthma is related to gut microbiota (23). The research discovered that *Lachnospira, Veillonella, Faecalibacterium*, and *Rothia*were reduced in stool samples from 3-month-old Canadian children with a high risk of asthma. Moreover, functional analysis of the bacterial community showed that the LPS pathway of biosynthesis was reduced across the microbiota of children at high risk of asthma (24). The study found the composition of *Proteobacteria, Bacteroidetes*, and *Actinobacteria* in allergic rhinitis were decreased (25). Thus, regulating gut microbiota may be an effective way to treat AA and AR.

Fecal microbiota transplantation (FMT) can be used to treat inflammatory bowel disease by reshaping the gut microbiota (26). Probiotics are microorganisms that promote human health by improving the intestinal micro-ecological balance, another choice to restore disorders of the microbiota may be probiotics. Interaction between a healthy microbiota and the immune system led to a well-balanced body (27). A dysfunctional microbiome and lack of diversity can lead to the evolution of disease (28). In this review, we discuss the characteristics of the microbiota in allergic airway inflammation (e.g., allergic rhinitis and asthma), the role of probiotics in experimental animal models and clinical studies.

## Methods

In order to discuss the effect of probiotics on respiratory tract allergic disease and gut microbiota, we undertook a systematized literature search that included observational studies (cross-sectional, cohort, or case-control) and experimental studies. The following exclusion criteria were used to reduce possible relationships observed due to other comorbidities: Atopic dermatitis, food allergies, intestinal disorders, respiratory microbiota. Searches were conducted using PubMed, Google Scholar, and Medline using the following key terms: allergic rhinitis OR asthma AND probiotics, allergic airway inflammation AND immune disorders, probiotics OR gut microbiota AND allergic disease, probiotics AND inflammatory. Studies from all years were included, specifically those published within the last 10 years. Some review articles and their reference lists were also searched to identify related articles. The search was limited to *in vivo* studies.

## Allergic Asthma

AA is the most commonly diagnosed respiratory allergic disease in clinical trials. It is a disease of chronic inflammation of the airways that involves multiple cells and cellular components such as mast cells, eosinophils, and lymphocytes. Its pathophysiology is complex, and many aspects are not well understood. Asthma is a critical global health problem which affects people of all ages. Its prevalence is growing in many countries, especially among children. Despite decreases in hospitalizations and asthma deaths in some countries, asthma still places an unacceptable burden on the health care system and society, especially the devastating effects of childhood asthma on families (29).

The disease affects about 300 million people worldwide and could rise to 400 million by 2025, influencing 15 percent of the world's children and adolescents (30). Asthma is caused by a combination of poorly understood genetic and environmental factors. Studies have systematically mapped the effects of single nucleotide polymorphisms (SNPs) on the presence of childhood onset asthma by genome-wide association. Characterized more than 317,000 SNPs in DNA from 994 patients with childhood onset asthma and 1,243 non-asthmatics, using family and case-referent panels, confirmed that Genetic variants regulating ORMDL3 expression contribute to the risk of childhood asthma (31). There is an association between IL33, IL1RL1/IL18R1, HLA-DQ, SMAD3, and IL2RB9 gene polymorphisms and the locus on chromosome 17q21 including ZPBP2, GSDMB, and ORMDL3 genes (32). In addition, these genes showed that abnormal epithelial barrier function, as well as innate and adaptive immune responses, can cause asthma.

After an allergic response and consequent stimulation in the presence of dendritic cells (e.g., epithelial-derived thymic stromal lymphopoietin and other coactivators) adaptive T helper 2 cells produce interleukin (IL)-5, IL-4, and IL-13. In addition, some patients with predominantly neutrophilic disease from T helper 1 (Th1) cell (IL-2, interferon, tumor necrosis factor), T helper 17 (Th17) cells (IL-17), 21 or type 3 innate cytokine release from Th1 cell, Th17 cells (33), or three innate lymphocytes, as well as macrophage activation and the release of neutrophil chemokines, such as the C-X-C motif chemokine ligand eight (7). High morbidity and mortality as asthma account for one in 250 deaths worldwide. Currently, hormone therapy such as inhaled corticosteroids, oral corticosteroids or glucocorticolds are used to treat asthma, but it is not effective in treating the root cause, and the heavy medical costs can be financially stressful. Multiple large-scale cluster analyses of asthma patients have identified various asthma endotypes, the incidence of which depends on age, gender, BMI, or inflammation spectrum of asthma onset (34–36). In these groups, early-onset asthma patients (asthma that occurs between 0 and 12 years of age) usually exhibit atopic symptoms, predominantly male; however, after puberty, the population is predominantly female (37). Therefore, it is essential to improve knowledge in this field to discover new treatments. Probiotics are live microorganisms that have proven to be beneficial to human health. Many clinical trials have demonstrated that probiotics can modulate the gut microbiota and that the gut microbiota can regulate the systemic immune system response and play an important role in the development of asthma, thus promoting the health of the body (27).

The onset of probiotic protection dates from fetal life (38), probiotic bacteria settled in the gut wield an influence on respiratory allergies and can relieve the symptoms. Supplementation with probiotics in pre-and postnatal periods was likely to play an essential strategic role in the prevention of asthma (39). Therefore, probiotic supplementation may be a new way to prevent/treat allergic diseases.

## Allergic Rhinitis

The prevalence of chronic inflammatory diseases of the upper respiratory tract, such as AR and chronic rhinosinusitis (CRS), often occurring in conjunction with asthma and conjunctivitis which affects up to 50% of people in some countries (40), it is a global health problem with a major burden worldwide. Indeed, AR lead to unproductive time at work, sleep problems, and for children it also reduces participation in outdoor activities (41–43). Clinical symptoms of AR include nasal bleeding, sneezing, nasal congestion, itching, burning or redness of the eyes, and an itchy throat. AR is known as IgE immunoglobulins-mediated disease, and the main disease-modifying treatment is allergen immunotherapy. These immune responses involve inflammation of the mucosa controlled by type 2 cells (44). Available treatments include avoidance of allergens, H1-antihistamines or intranasal corticosteroids drug therapy, and allergen-specific immunotherapy (AIT) (45). However, the use of drugs for allergic rhinitis (such as histamine antagonists) can produce undesirable side effects, such as fatigue. Probiotics may be a new treatment for allergic rhinitis (46).

Probiotic interventions are potentially useful with low adverse effects such as *Lactobacillus* and *Bifidobacterium*. The consumption of probiotics may provide a more balanced gut microbiota in people with allergic rhinitis, which may limit the damage caused by inflammation. In addition, well-balanced gut microbiota may lead to less severe reactions to allergens (47). However, further research is needed to fully understand the potential mechanisms. The preventive effects of probiotics on allergic diseases have been reported, and although there was considerable variation between these studies, the results suggested that probiotics have important clinical and immunological effects in the treatment of allergic rhinitis (48).

## Commensal Gut Microbiota in Early Life Shapes the Immune System

The gut is the main site of interaction between the host immune system and both commensal, as well as pathogenic microbes during the first years of life. Immunity is shaped by commensal microbiota. From early life onwards, microbes colonize mucosal surfaces of the body and thereby trigger the establishment of immune homeostasis and defense mechanisms (49). Evidence reveals that the family of innate lymphoid cells (ILCs), which are mainly located in mucosal tissues, are essential in the maintenance of barrier functions as well as in the initiation of an appropriate immune response upon pathogenic infection. The formation of early lymphoid tissue inducer cells (LTi) seems to be independent of the presence of the commensal microbiota as germ-free (GF) mice harbor normal numbers of lymph nodes and Peyer's patches (50). Maternal dietary components, such as retinoic acid (RA), were shown to be crucial to maintain this LTi cell population during fetal development. Microbiota is directly regulating the availability of RA (51). CCR6^+^c-kit^hi^ LTi cells seed the intestinal lamina propria during fetal development, and low numbers of CCR6^−^c-kit^lo^ ILC3s that expand strongly within the first 2–4 weeks of birth and that can acquire NK cell markers (52), which was shown to be dependent on signals originating from the maternal microbiota as shown in a model of reversible gestational colonization during pregnancy (53). Aryl hydrocarbon receptor (AhR) ligands produced by the maternal microbiota were transferred to the offspring postnatally through the milk and permanently increased the absolute number of NKp46^+^ILC3s in the offspring small intestinal lamina propria starting at postnatal day 14 (54). However, subject to controversy as other studies showed a negative influence of missing microbiota on the different ILC3 subsets (55). Natural ligands of the AhR include agonists derived from cruciferous vegetables in the diet as well as indoles produced by members of the microbiota. It is likely to speculate that AhR ligands derived from not only the maternal microbiota but also from the endogenous microbiota of the offspring contribute to the homeostasis of this ILC population. While the T-bet induced upregulation of NK cell receptors (NCRs) on ILC3s was dependent on the presence of the commensal microbiota as described above (56) the loss of RORyt expression in NCR^+^ILC3s and their switch to a more ILC1-like phenotype was prevented in colonized compared with GF mice (54). The role of microbiota in the differentiation of ILC1s and NK cells during early life has not been investigated in detail. A study suggested that colonization of the neonatal intestine is triggered through microbiota colonization after birth as this subset seems absent in the fetal intestine. In addition, they show that this ILC1 subset has the potential to differentiate into RORγt-ex- pressing ILC3 during adulthood in the presence of IL-23 and RA, the latter being partially regulated by the commensal microbiota (57). ILC1 and ILC2 subsets were most affected by the absence of commensal microbiota and acquired a phenotype that more closely resembled ILC3 subsets. In conclusion, while ILC3s seem to be mainly influenced by microbial signals during early life, ILC2s and ILC1s are more shaped through microbiota during adulthood.

Microbiota-induced IL-1β also induces the release of granulocyte monocyte colony-stimulating factor (GM-CSF) by ILC3, which in turn promotes mononuclear phagocytes to produce regulatory components, such as IL-10 and RA (58). The latter are important to promote regulatory T-cell (Treg) differentiation and expansion in the intestine to ensure intestinal homeostasis. In the absence of microbiota that functionally impaired DCs were unable to produce the NK priming cytokines IFN-I and IL-15. Behave as splenic NK cells in GF and antibiotic-treated mice are largely unresponsive when stimulated to produce IFN-c or exert specific lysis of target cells following Toll-like receptor (TLR) ligand exposure *in vivo* (59).

While ILC function is influenced by commensal microbiota, ILCs themselves can influence adaptive immune cells, which in return help keeping the host-microbial mutualism in check. The elimination of microbiota-specific T-cells was very important to prevent low-grade systemic and spontaneous intestinal inflammation (60). An independent study demonstrated that splenic but not intestinal MHC-II-expressing ILC3s can be activated through microbiota-derived IL-1b to express co-stimulator molecules and thus drive CD4+T-cell and B-cell responses *in vivo* (61). While microbiota diversity and phylum composition were unaltered in the absence of ILC3s, these mice exhibited higher levels of segmented filamentous bacteria (SFB) as well as *Clostridiales* species (62). A study demonstrated that Id2 expression in ILC3s was important for the generation of IL-22, which maintained a healthy microbiota that exhibited early colonization resistance to *Citrobacter rodentium* (62).

## The Role of Gut Microbiota in Allergic Conditions

The gut microbiota is the normal microorganisms that colonize the human intestine are in a symbiotic relationship with the body and are diverse and abundant. There is evidence that more than 50 diseases are associated with dysbiosis of the gut microbiota, such as many infectious diseases, liver diseases, gastrointestinal malignancies, metabolic disorders, and allergic diseases (63). The colonization of gut microbiota early in life affects the immune status during childhood, and early gut microecological dysregulation due to various factors (mode of delivery, feeding practices, antibiotic use, environmental exposure, etc.) may disrupt the homeostatic regulatory mechanisms between Th1/Th2 cells, which in turn may negatively affect the development of immune tolerance and may eventually activate the allergic process, increase the risk of allergy, and thus induce and exacerbate allergic diseases (64).

The gut microbiota plays a crucial role in the maturation process of the postnatal immune system, especially in immune tolerance. It was found that germ-free mice failed to induce Th2-mediated immune tolerance, whereas oral immune tolerance developed after the re-establishment of gut microbiota. The role of gut microbiota on immune development is mediated by TLR2 through DCs. In addition, regulatory T cells play a key regulatory role in immune tolerance. The gut microbiota and its metabolites can induce regulatory T cells and participate in the formation of mucosal immune tolerance. The gut microbiota can also enhance the mucosal barrier effect by stimulating the secretion of sIgA. The gut microbiota protects the host from allergic reactions by acting on both intrinsic and adaptive immunity (65).

Disturbances in the gut microbiota also affect the development of immune tolerance in the respiratory mucosa. Gill et al. reported the relationship between pulmonary immunity, the mucosal immune system, and the gut microbiota group: changes in the gut microbiota lead to alterations in the downstream immune response, affecting the development of the immune system, which in turn affects the immune response in distal mucosal organs (e.g., the lung, which subsequently leads to the development of pulmonary inflammation); abnormalities in the immune system, In turn, affect the gut microbiota composition, which in turn affects the immune response of the distal mucosal system and triggers an immune imbalance in the lungs (66). The gut microbiota can enhance pulmonary immunity and clear pulmonary pathogens through the flora and its metabolites, reducing the development of pulmonary diseases; conversely, pulmonary diseases can affect the structural composition and diversity of the gut microbiota, causing the corresponding intestinal symptoms (67). Modern medicine refers to the interaction between the intestine and the lung as the “Gut-Lung axis”. The “Gut-Lung axis” helps us to better understand the relationship between gut microbiota and allergic diseases.

## Asthma and Allergic Rhinitis Alter Microbiota in the Gut

The human intestine starts from the mouth, passes through the esophagus, stomach, small intestine, colon, and finally reaches the rectum. Its huge surface area of 150–200 square meters provides an opportunity for microbes to colonize (4). They are mainly composed of the following five phyla: *Bacteroidetes, Firmicutes, Proteobacteria, Actinobacteria* (68, 69), and *Bacteroides, Faecalibacterium*, and *Bifidobacterium* are the most common genera in healthy adults (70). The bacterial composition of each part is as follows: the main colonizing bacteria of the oral cavity are: *Streptococcaceae, Pasteurellaceae, Veillonellaceae, Prevotellaeace*, and *Neisseriaceae* families and *Gemella* genus; the stomach mainly contains bacteria from the *Lactobacillaceae* family; the small intestine is dominated by *Lactobacillaceae, Enterobacteriaceae*, and *Streptococcaceae*; the large intestine contains *Enterococcaceae, Clostridiaceae, Enterobacteriaceae, Bacteroidaceae, Bifidobacteriaceae, Fusobacteriaceae, Lactobacillaceae, Peptostreptococcaceae, Peptococcaceae, Prevotellaeace, Lachnospiraceae, Ruminococcaceae, Rikenelleace*from families level, and the phylum *Verrucomimicrobia* (71, 72). Many functions of the host, including the production of vitamins, ion absorption, resistance to pathogens, histological development, immune function enhancement, and food fermentation all require the involvement of the gut microbiota (4).

Supplementation of feces from ovalbumin (OVA)-induced asthma mouse model to germ-free mice with representative species of the genera *Lachnospira, Veillonella, Faecalibacterium*, and *Rothia*which may induce airway inflammation (71). In another human birth cohort study, children with an increased relative abundance of *Streptococcus* and *Bacteroides* species and decrease *Bifidobacterium* species and *Ruminococcusgnavus* in fecal samples at 3 months of age have a higher risk of allergic reactions and wheezing at 5 years of age (6). Moreover, among American newborns classified into three groups based on gut microbiota composition, those with the lowest relative abundance of *Bifidobacteria, Akkermansia*, and *Faecalibacterium* and the highest relative abundance of *Candida* and *Rhodotorula* had the highest risk of developing asthma (71).

The effects of the gut microbiota on asthma are mediated, at least in part, by bacterial metabolites that may influence immune responses at distal sites in the body. The best-known metabolite is short-chain fatty acids (SCFA), which has protective properties in inflammation of the human respiratory tract. Children with high fecal butyrate and propionate levels at 1 year of age are significantly less likely to have atopic allergies and less likely to develop asthma at 3–6 years of age (24). In mice, SCFAs have been shown to increase the expression of the transcription factor FOXP3 by inhibiting histone deacetylation, thereby promoting the proliferation of Tregs and increasing IL-10 production (73). SCFAs have also been shown to be anti-inflammatory in models of airway inflammation induced by OVA and house dust mites (HDM) (74, 75). Recent studies have shown that human gut microbiota are capable of producing other pro and anti-inflammatory potential metabolites, like biogenic amines (including histamine) (76) and oxylipins such as 12, 13-diHOME (77). The numbers of histamine-secreting bacteria were significantly higher in asthma patients' fecal samples than non-asthmatic volunteers (78). Furthermore, the number of histamine-producing bacteria correlates with the severity of the disease. However, bacterial-derived histamine reduced the total number of cells in bronchoalveolar fluid (BAL) and the amount of IL-4, IL-5 and IL-13 in lung homogenates in an OVA-induced allergic airway inflammation model (79), illustrating the complexity of bacterial immune regulation. In contrast, intra-abdominal treatment of mice with 12, 13-diHOME reduced the number of Treg cells in the lung and increased lung inflammation in a cockroach antigen mouse model of airway inflammation (79). Metabolites secreted by gut microbiota may prove useful for other treatments.

In allergic rhinitis, the gut microbiota is emerging as a new target for early intervention in childhood atopic diseases. Recent studies suggested that a higher bacterial ratio between *Klebsiella* (an opportunistic pathogen) and *Bifidobacterium* (commensal bacteria of the gut microbiota) may predispose to allergic diseases (80), further studies suggest that the use of probiotics in infants may favorably alter this ratio and may prevent the future development of allergic diseases (81). Although the study did not find a significant correlation between first-year antibiotics and allergic rhinitis, it did find a significant correlation between first-year antibiotics and asthma, which is usually associated with allergic rhinitis (81).

High-throughput sequencing of bacteria in the fecal of 93 AR patients revealed that the diversity of the gut microbiota of AR patients was significantly reduced, with an increased relative abundance of *Bacteroidetes* at the phylum level and decreased relative abundance of *Actinobacteria* and *Proteobacteria* at the genus level, and significantly decreased relative abundance of *Escherichia-Shigella*. An analysis of LefSe showed that *Escherichia-Shigella, Lachnoclostridium, Parabacteroides* and *Dialister* were potential biomarkers of AR (82). Shannon index analysis showed a significant reduction in species richness and a reduction in chao1 diversity indices in AR patients. In the AR group, *Bacteroidetes* were significantly abundant. In contrast, the *Firmicutes* phylum was significantly less abundant. In addition, the abundance of *Parabacteroides* increased and the abundance of *Oxalobacter* and *Clostridiales*decreased in AR adults (83). Another adult study showed that the AR group had significantly higher bacterial diversity than the non-AR group, *Firmicutes, Fusobacteria, Actinobacteria, Cyanobacteria*, and *Chloroflexi*were the significantly differed phyla. Although bacterial diversity showed no remarkable differences between patients with moderate and severe AR, there was a significant correlation between nasal symptom scores or rhinopharyngitis levels and *Butyricoccus* and *Eisenbergiella* levels, revealing the potential for intervention through gut microbiota (84). Changes in the gut microbiota in allergic asthma and allergic rhinitis showed in Table 1.

**Table 1:** Changes of the gut microbiota in allergic asthma and allergic rhinitis.

**Type of disease**	**Increase**	**Decrease**
		*Lachnospira*
		*Veillonella*
		*Faecalibacterium*
	*Streptococcus*	*Rothia*
	*Firmicutes*	*Bifidobacterium*
Allergic asthma	*Candida*	*Ruminococcusgnavus*
	*Rhodotorula*	*Akkermansia*
		*Bacteroidetes*
		*Alloprevotella*
		*Oscillibacter*
		*Lactobacillus*
		*Bifidobacterium*
	*Klebsiella*	*Actinobacteria*
	*Parabacteroides*	*Escherichia-Shigella*
Allergic rhinitis	*Butyricicoccus*	*Oxalobacter*
	Proteobacteria	*Clostridiales*
		*Eisenbergiella*
		*Bacteroidetes*

Therefore, ameliorating AR through modulating gut microbiota may be a potential way, but the specific mechanism needs further study.

## Probiotics and Their Potential Benefits

Probiotics are defined as “live microorganisms” that have a positive effect on host health when administered in sufficient quantities. It was discovered that the gut microbiota can be altered by oral beneficial bacteria and replace the harmful microorganisms, thus producing the concept of probiotics (85). Probiotics colonize and multiply in the intestine, enhance epithelial integrity, attach to the intestinal epithelium, increase adhesion to the intestinal mucosa, compete to exclude pathogenic microorganisms, resist the production of bactericidal substances, and maintain the gut microbiome's ecological balance (86).

Probiotics exhibited multiple mechanisms: probiotics can activate or inhibit type 1 T helper cells through altering the composition of the gut microbiota; probiotics are also stimulating interleukin 10, which suppresses the inflammatory response (87). In addition, probiotics may decrease an antigen-specific IgE levels in serum (88). Several probiotic strains, such as *Lactobacillus rhamnosus* GG, *Streptococcus thermophilus, Lactobacillus plantarum*MB452, and the gram-negative probiotic strain *Escherichia coli Nissle* 1917 was shown to increase the integrity of the epithelial barrier by enhancing tight junction-related genes or adhesion junction-related receptors through their microbiologically relevant molecular patterns with pattern recognition receptors in epithelial cell gene expression (89–91). In addition, it is essential that probiotic strains must be safe and effective for humans, maintain activity for the shelf life of the product, and not be pathogenic (92).

Research has shown that a particular probiotic strain has immunomodulatory functions, and can alleviate the symptoms of allergic airway inflammation (93). Clinical and laboratory evidence shows that there is a therapeutic effect of probiotics on allergic diseases by regulating gut microbiota and modulating host immunity, promoting the maintenance of normal immune tolerance (94).

## Regulatory Mechanism of Probiotics

The increasing evidence of a relationship between alterations in the microbiota and asthma supports the idea that the microbiota could be harnessed to treat allergic airway inflammation. Probiotics and gut microbiota interact and confer an array of positive effects on the epithelial layer to maintain gastrointestinal and systemic health, by interacting with the gut-associated lymphoid tissues (GALT) which mediate oral tolerance and mucosal immunity (95). Both probiotics and commensal bacteria enforce the functions of the mucosal barrier of the GIT epithelia, induce mucus secretion, and stimulate secretion of IgA which neutralizes pathogens inside the lumen (96). Correspondingly, cross-talk between epithelial cells and residing epithelial immune cells are mediated and enforced by probiotics, and contribute to their effector functions (97). Probiotics are also able to induce the expression of adhesion molecules, stimulate the innate immune system, antigen-presenting cells (APC) and natural killer (NK) cells in both mice and humans (98). Adaptive immunity is stimulated by probiotics, such that IgG and IgA antibodies are produced in response to probiotic consumption (99). In addition, probiotics can stimulate CD8+ T cells, Treg cells and cytokines (i.e., interferon (IFN)-gamma and IL-10 (100). APCs exposed to probiotics present harmless peptides to T cells and subsequently induce Treg cells, to produce anti-inflammatory cytokines including, transforming growth factor beta (TGF-β), IL-10 and retinoic acid. In addition, Treg cells suppress effector Th1, Th17 and cytotoxic T (Tc) cells and IgA secretion (101). Thus, probiotic bacteria can control the “on/off” switch of immune responses in a strain-dependant manner, modulating the host immune system at the mucosal level. Lacking of sufficient probiotic bacteria and their subsequent stimulatory impact on the immune system leads to inadequate or inappropriate immune modulation. Insufficient probiotic bacteria alone, or inadequacy, together with stimulation of the immune system by invasive pathogens, mediate a range of immunopathogenic disorders such as AA and AR.

## Allergic Asthma

### Animal Studies

#### Modulation of Allergic Asthma and Its Gut Microbiota by Probiotics

Probiotics confer immunological protection to the host through the regulation, stimulation, and modulation of immune responses, and have been widely promoted due to their positive effect on the attenuation of abnormal immune responses such as asthma. The phenotype of allergic airway disease is influenced by host genetics and interactions between the gut microbiota, may be regulated by probiotics. The phenotype of allergic airway disease is influenced by host genetics and interactions between the gut microbiota, the gut microbiota may be regulated by probiotics.

The study gavaged *Lactobacillus paracasei* L9 for OVA-induced acute asthma mice, and found that *Lactobacillus paracasei* L9 reduced airway hyperresponsiveness and the proportion of eosinophils in bronchoalveolar lavage fluid by correcting the Th2/Th1 immune imbalance in the lungs of mice, thereby alleviating asthma (102). In another study, oral administration of *Lactobacilli* to asthmatic rats, resulted in decreased levels of inflammatory infiltrates (IL-4, IL-5), eosinophils, and serum allergen-specific IgE, IgA, TGF-β, mucin-2, and tight junction protein were increased, and intestinal morphological changes were normalized (103).

In addition, administration of *Bifidobacterium breve* M-16V (*B. breve* M-16V) to pregnant mice exposed to residual oil fly ash also resulted in a reduction in eosinophils in bronchoalveolar lavage fluid and reduced allergic lung inflammation in neonatal mice, besides decreased expression of Th2 cytokines (IL-5 and IL-13). Also, oral administration of *B. breve* M-16V reduced *Firmicutesin*. Besides, several bacterial strains of the mouse fecal microbiota were strongly associated with Th2 cytokine and histological scores (104). Moreover, studies using *Bifidobacterium longum* 7952 (*Bl*7952) in OVA-sensitized mice showed allergy-reducing properties and inhibition of airway inflammation, as well as reduced eosinophil levels in bronchoalveolar lavage fluid (BALF). Systemic and local levels of Th2-related cytokines and OVA-specific IgE antibodies were decreased by Bl7952 (105). The oral administration of *Bifidobacterium longum* 51A to both male A/J and C57BL/6 mice with airway inflammation revealed that only the A/J mice showed reduced inflammation, which was partly due to acetate production. Acetate-producer probiotics modulated the abundance of specific bacterial (e.g., *Akkermansia* and *Allistipes*) genera in the mouse strain. The gut microbiota diversity was increased in C57BL/6 mice given probiotics compared to A/J mice at baseline. To understand the relationship between microbial composition and the development of allergic disease, implantation of A/J embryos into female C57BL/6 mice showed an increased diversity of the gut microbiota and a decreased eosinophilic inflammation in A/J mice (106). Furthermore, oral administration of *Feacalibacterium prausnitzii* to house dust mite-induced asthma mice reduced inflammatory cell infiltration and cytokine secretion, alleviated pathological changes, and increased the proportion of Treg. It improved microbial ecological dysregulation and enhanced SCFA production and raised *Faecalibaculum, Dubosiella, Streptococcus*, and *Lachnoclostridium* abundance. It also normalized pathways involved in carbohydrate and lipid metabolism, which may relate to SCFA production. Besides correlation analysis showed that immune indicators are closely related to the production of SCFA (107). Oral administration of *Lactobacillus rhamnosus* diminished the characteristics of allergic asthma in a mouse model and induced immunomodulatory effects by a CD4 (+) CD25 (+) Foxp3 (+) Treg cell-mediated mechanism (108). Oral administration of *Lactobacillus rhamnosus* GG prevents OVA-induced allergic airway inflammation by amplifying mesenteric CD103+ DCs and accumulating mucosal Tregs cells. The diversity of the gut microbiota was increased, the Shannon and Simpson index and the abundance of *Bacteroidetes, Alloprevotella*, and *Oscillibacter* increased, while the abundance of *Firmicutes* decreased (109). Thus, single probiotics play a beneficial role in the prevention of allergic diseases.

Probiotic-induced protection is associated with gut microbiota and provides new evidence for the use of probiotics in allergic airway inflammation. The association between allergic asthma and microbiota varies by diet and has been extensively studied, implying the potential of oral alternative medicine supplements for the control of asthma. According to Song et al. three probiotic mixtures (*Lactobacillus plantarum* GREEN CROSS Wellbeing 1001, *Lactobacillus rhamnosus* GCWB 1,085, and *Lactobacillus rhamnosus* GCWB 1,156) were orally administered to mice in a diesel exhaust-induced BALB/c asthma model to reduce inflammatory cell levels. In addition, IgE and matrix metalloproteinase (MMP)-9 activities was significantly reduced in serum and BAL (110). In another study, six mixed probiotics (*Lactobacillus gasseri* LK001; *Lactobacillus salivarius* LK002; *Lactobacillus johnsonii* LK003; *Lactobacillus paracasei* LK004; *Lactobacillus reuteri* LK005, and *Bifidobacterium animals* LK011) alleviate OVA-induced asthma in mice by reducing irritant DC surface moleculesand increasing CD103+ DC-induced mucosal irritant Treg in the mesentery (111). Probiotics enhanced gut microbial diversity and *Bacteroidetes/Firmicutes* (*B/F*) ratios, and increased levels of *Lactobacillus* in mice (112).

Therefore, targeting the gut microbiota would be an effective approach to treating allergic airway diseases. Mixed probiotics are of particular interest for their beneficial effects on the host, but their potential mechanisms need to be further explored. There are fewer studies of mixed probiotics in mice and more research is needed to confirm their beneficial effects in asthma treatment.

### Human Studies

**Figure 1:**
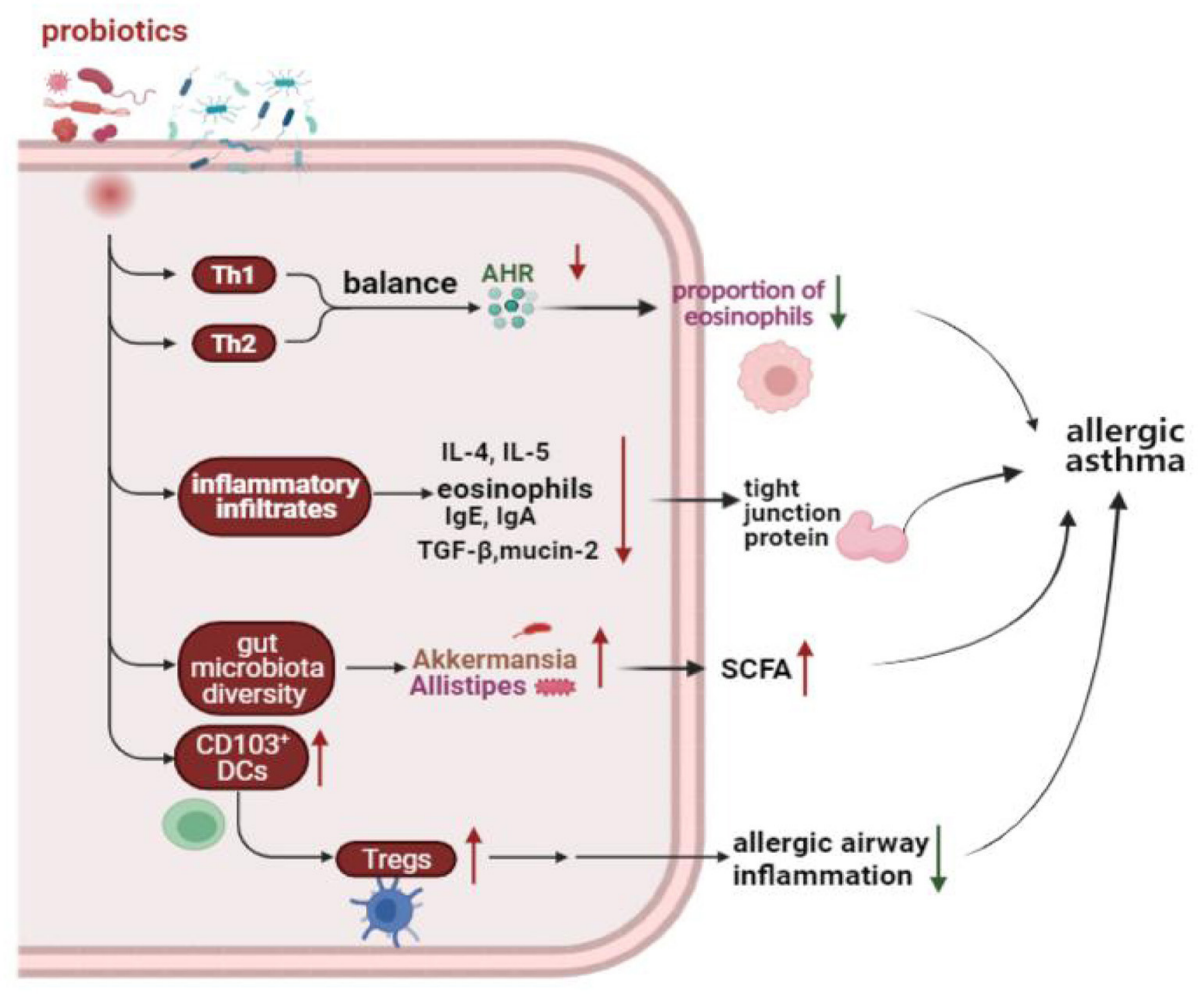
Effect of probiotics on allergic asthma. Probiotics protect the homeostasis of the immune system by regulating the Th1 and Th2 balance, reducing the inflammatory response, increasing the number of Tregs, and modulating the gut microbiota. Th1, T helper 1; Th2, T helper 2; AHR, Airway hyperresponsiveness; IgE, Immunoglobulin E; IgA, Immunoglobulin A; IL-4, Interleukin 4; IL-5, Interleukin 5; TGF-β, Transforming growth factor-beta; DCs, Dendritic cells; Tregs, Regulatory T cells; SCFA, Short-chain-fatty acids.

A meta-analysis of randomized controlled trials (RCTs) showed that supplemented with *Lactobacillus rhamnosus* GG reduced the incidence of asthma, and probiotic supplementation before and after delivery may play an important strategic role in asthma prevention (113). However, a randomized, controlled, double-blind study of 159 newborns found that supplementation with *Lactobacillus rhamnosus* GG for the first 6 months of life seems not to prevent the development of asthma at age two (114). In addition, using of probiotics for asthma early in life demonstrated a significant reduction intotal IgE and atopic allergy in a meta-analysis (115). Moreover, patients with asthma have elevated blood levels of TNF-α, interferon-δ, and IL-12. Chen et al. found there was a positive effect on clinical symptoms and cytokine levels in asthmatic children 6 to 12 years old on daily doses of *Lactobacillus* for 2 months (39). In four meta-analyses of RCTs, probiotics had no benefit for asthma treatment (116). The use of probiotics in the clinical treatment of asthma is still controversial, and a large number of clinical studies are still needed to confirm their effectiveness. Effect of probiotics on allergic asthma showed in Figure 1.

## Allergic Rhinitis

### Animal Studies

#### The Effect of Probiotics on the Regulation of Allergic Rhinitis and Its Gut Microbiota

Ren et al. investigated the benefits of oral administration of *Bifidobacterium* short (*B. breve*) in allergic rhinitis and observed that *B. breve* exert its anti-allergic function by suppressing type II helper T cell immune responses and enhancing CD4+CD25+Treg activity. Allergic rhinitis symptoms such as sneezing were reduced when *B. breve* was administered at doses of 10^7^ CFU or higher (117). Cui et al. found that oral administration of *Lactobacillus Plantarum* CJLP133 or CJLP243 in a mice model of AR improved allergic rhinitis by reducing airway hyperreactivity and histological scores, as well as the number of infiltrating cells in the nasal cavity and lungs. Samples of bronchoalveolar lavage fluid and draining lymph nodes from mice showed reduced immune cell counts and increased secretion of Th1 and Th2 type cytokines, decreased IL-4, IL-5, IL-13, serum IgE, and specific serum IgG1 levels, and increased secretion of IFN-γ and specific serum IgG2a (118). Our research in oral administration of *Clostridium butyricum* (*C. butyricum*) CGMCC0313-1 which reduced allergic airway inflammation induced by ovalbumin in mice, and found *C. butyricum*significantly reduced lung resistance, pulmonary airway inflammation, mast cell degranulation, airway remodeling, and OVA-specific IgE/G1 expression in asthmatic mice. In addition, it reversed the Th1/Th2 imbalance and increased the anti-inflammatory cytokine IL-10 (119).

Kim et al. revealed that treatment with a probiotic mixture (PM) of *Bifidobacterium longum* IM55 and *Lactobacillus Plantarum* IM55 isolated from human feces and kimchi could alleviate AR by restoring Th2/Treg imbalance (inhibiting Th cell differentiation to Th2 cells and inducing differentiation to Treg cells) and gut microbiota disorders (significantly suppressing *Proteobacteria* and increasing the composition of *Bacteroidetes* and *Actinobacteria*) (120).

### Human Studies

A randomized, double-blind, placebo-controlled study in 40 children worldwide discovered significant improvements in allergic rhinitis symptoms and quality of life in children treated with a probiotic mixture (*Bifidobacterium longum* BB536, *Bifidobacterium infantis* M-63, *Bifidobacterium shortum* M-16V), all parameters significantly better than the placebo group (121). Similarly, in a double-blind, placebo-controlled, parallel, randomized clinical trial study, Dennis-Wall et al. suggested that the ingestion of *Lactobacillus garciae*KS-13, *Bifidobacterium bifidum* G9-1, and *Bifidobacterium longum* MM-2 improved the overall scores of patients with rhinoconjunctivitis on the quality of life questionnaire, the percentage of Tregs in fasting whole-blood samples increased. However, the mechanisms involved are not clear (87).

**Figure 2:**
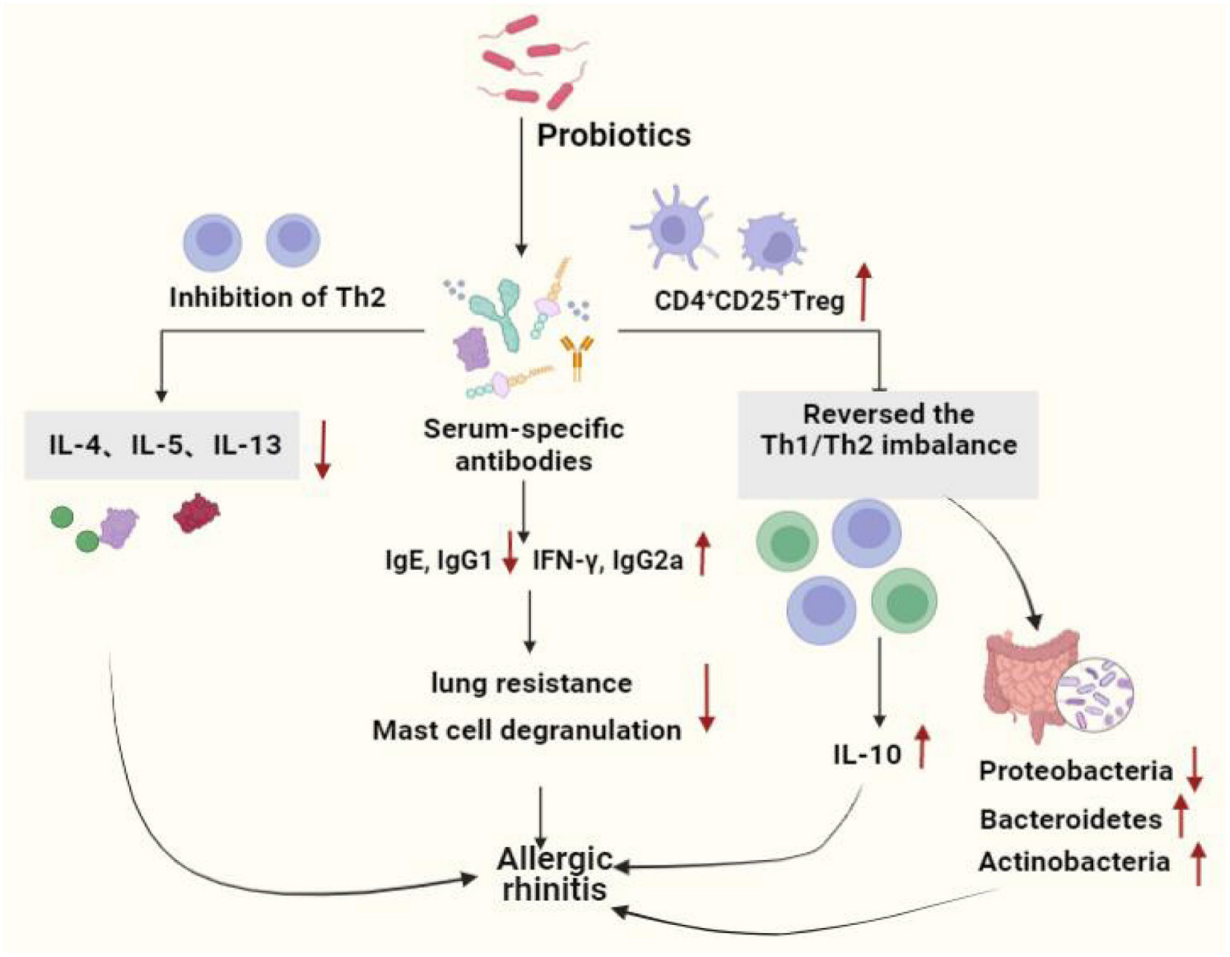
Effect of probiotics on allergic rhinitis. Probiotics protect the allergic rhinitis by decreasing inflammation in serum and lung tissue, increasing the number of immune cells, immune system homeostasis by regulating the Th1 and Th2 balance and increasing the number of Tregs. Moreover, probiotics regulated intestinal stability by increasing the level of beneficial bacteria, hence improving AR. Th1, T helper 1; Th2, T helper 2; IL-4, Interleukin 4; IL-5, Interleukin 5;IL-10, Interleukin 10;IL-13, Interleukin13; IgG1, Immunoglobulin G1; IgE, Immunoglobulin E; IgG2a, Immunoglobulin G2a; IFN-γ, Interferon-γ.

A prospective trial study found that adjuvant conventional treatment with *Enterococcus faecalis* reduced the number and duration of rhinitis attacks in children and adolescents (122). Ye et al. systematically reviewed and meta-analyzed the therapeutic effects of probiotics on AR, there were 16 clinical trials (randomized controlled trials), 1,374 patients involved, and found that probiotics were highly effective in reducing symptoms in patients with AR compared to the placebo group (123). Ivory et al. showed that volunteers treated with *Lactobacillus casei Shirota* (LcS) reduced antigen-induced production of cytokines show that probiotic supplementation modulates immune responses in AR and may have the potential to alleviate the severity of symptoms (124). However, the same group Ivory et al. in 2013 showed that changes of the immune status did not have any effects on the clinical symptoms (125). Lack of changes in the clinical symptoms could be explained by an inability of a single nasal allergen challenge to reproduce the chronicity of a natural allergen exposure (126), it does not represent a real-life situation where individuals are exposed to lower concentrations of allergen over a prolonged period of time. Both allergen dose and multiplicity of challenge can influence the outcome. Effect of probiotics on allergic rhinitis showed in Figure 2. Probiotics used in allergic asthma and allergic rhinitis showed in Table 2.

**Table 2:** Probiotics use in allergic asthma and allergic rhinitis.

**Type of diseases**	**Probiotics**	
	**Single probiotics**	**Mix probiotics**
	*Lactobacillus paracasei L9*	
	*Lactobacilli*	
	*Bifidobacteriumbreve* M-16V Bifidobacteriumlongum7952	
Allergic asthma	*Bifidobacteriumlongum 5*1A	*Lactobacillus plantarum GREEN CROSS Wellbeing 1001, Lactobacillus rhamnosus GCWB1085, and Lactobacillus rhamnosus GCWB 1156*
	*Feacalibacteriumprausnitzii*	*Lactobacillus gasseri LK001, Lactobacillus salivarius LK002, Lactobacillus johnsonii LK003, Lactobacillus paracasei LK004, Lactobacillus reuteri LK005, and Bifidobacterium animals LK011*
	*Lactobacillus rhamnosus*	
	*Lactobacillus rhamnosus GG*	
	*Lactobacillus*	
	*Bifidobacteriumshortum*	
	*Lactobacillus plantarum CJLP133 or CJLP243*	
Allergic rhinitis	*Clostridium butyricum CGMCC0313-1*	*Bifidobacteriumlongum BB536, Bifidobacteriuminfantis M-63, Bifidobacteriumshortum M-16V*
	*Bifidobacteriumlongum IM55*	*Lactobacillus garciae KS-13, Bifidobacteriumbifidum G9-1 and Bifidobacteriumlongum MM-2*
	*Lactobacillus plantarum IM55*	
	*Enterococcus faecalis*	

## Conclusions

In conclusion, this study summarized the role of probiotics as an intervention or treatment for common allergic diseases. Probiotics reduced allergen-induced hyperreactivity and inflammation as well as reduced cytokine release, among other beneficial effects. In several studies, no significant differences were found between the probiotic-treated and placebo-treated. Although probiotics did not eliminate allergies, their administration may reduce the incidence and duration of allergy symptoms. However, the effects of probiotics depend on their species or strain, their derived metabolites, and the patient's gut microbiota. This review provided a broader understanding of new alternatives for the treatment of allergies, in both pediatric patients and adults, showing that probiotics can reduce symptoms in some cases as well as the severity of such disorders. However, there are limitations to some studies, such as small sample sizes, randomization processes, and the use of small numbers of bacterial populations.

Although numerous studies have been conducted, future basic and human studies that must be done to solidify the role of probiotics in treating allergies. Most current studies have found preliminary effects of probiotics in the treatment of allergic diseases in the clinic. However, some studies have found that probiotic treatment did not significantly improve clinical symptoms, possibly because clinical trials include many variables such as concomitant disease, age, gender and many other factors. Inclusion of patients and standardization of probiotic administration should be standardized in future studies to provide sound evidence and detailed information for the beneficial effects of probiotics. In conclusion, the administration of probiotics in respiratory allergic diseases seems to be promising. In addition, probiotics as a vehicle to reshape the gut microbiota may be a new therapy in the future.

## Author Contributions

JLH conducted the literature search and wrote the first draft of the manuscript. XS and JZ reviewed and XZW, ZHJ, and PPZ commented on several drafts. All authors have read and agree to the published version of the manuscript.

## Funding

This work was supported by the National Natural Science Foundation of China (82170026) and the Discipline Promotion Project of Xijing Hospital (XJZT18MJ23).

## Conflict of Interest

The authors declare that the research was conducted in the absence of any commercial or financial relationships that could be construed as a potential conflict of interest.

## Publisher's Note

All claims expressed in this article are solely those of the authors and do not necessarily represent those of their affiliated organizations, or those of the publisher, the editors and the reviewers. Any product that may be evaluated in this article, or claim that may be made by its manufacturer, is not guaranteed or endorsed by the publisher.
